# The Protective Effect of Fasudil on the Structure and Function of Cardiac Mitochondria from Rats with Type 2 Diabetes Induced by Streptozotocin with a High-Fat Diet Is Mediated by the Attenuation of Oxidative Stress

**DOI:** 10.1155/2013/430791

**Published:** 2013-05-22

**Authors:** Rong Guo, Baoxin Liu, Shunping Zhou, Buchun Zhang, Yawei Xu

**Affiliations:** Department of Cardiology, Shanghai Tenth People's Hospital, Tongji University School of Medicine, 301 Yan Chang Zhong Road, Shanghai 200072, China

## Abstract

Dysfunction of cardiac mitochondria appears to play a substantial role in cardiomyopathy or myocardial dysfunction and is a promising therapeutic target for many cardiovascular diseases. We investigated the effect of the Rho/Rho-associated protein kinase (ROCK) inhibitor fasudil on cardiac mitochondria from rats in which diabetes was induced by a combination of streptozotocin (STZ) and a sustained high-fat diet. Eight weeks after diabetes was induced by a single intraperitoneal injection of 50 mg/kg STZ followed by a sustained high-fat diet, either fasudil (5 mg/kg bid) or equivalent volumes of saline (control) were administered over four weeks. Fasudil significantly protected against the histopathologic changes of cardiac mitochondria in diabetic rats. Fasudil significantly reduced the abundances of the Rho A, ROCK 1, and ROCK 2 proteins, restored the activities of succinate dehydrogenase (SDH) and monoamine oxidase (MAO) in cardiac mitochondria, inhibited the opening of the mitochondrial permeability transition pore, and decreased the total antioxidant capacity, as well as levels of malonyldialdehyde, hydroxy radical, reduced glutathione, and superoxide dismutase in heart. Fasudil improved the structures of cardiac mitochondria and increased both SDH and MAO activities in cardiac mitochondria. These beneficial effects may be associated with the attenuation of oxidative stress caused by fasudil treatment.

## 1. Introduction

Mitochondria are recognized as essential cell organelles, which generate most of the cell's energy. In addition, mitochondria are involved in many physiological activities such as cell signaling, proliferation, growth, and death [1]. They have been implicated in cardiac dysfunction and myocardiocyte damage by the loss of metabolic capacity and the production or release of toxic substances [2]. Therefore, mitochondria are regarded as a novel therapeutic target in ischemic heart disease and some cardiomyopathies [3].

Diabetes mellitus (DM) is a major cause of serious microvascular and macrovascular diseases, affecting nearly every system in the body. Elevated oxidative stress in diabetic patients and in animal models of diabetes results from overproduction of reactive oxygen species (ROS) and decreased efficiency of antioxidant defenses [4, 5]. Moreover, diabetes-associated metabolic disorders and glycated or oxidized low-density lipoproteins (ox-LDL) impair the activities of enzymes of the mitochondrial respiratory chain complex [6]. Therefore, oxidative stress is closely related to mitochondrial dysfunction.

Rho-associated kinases (ROCKs) seem to contribute to numerous pathophysiological pathways that are triggered by hyperglycemia and represent promising molecular targets for cardioprotective treatment [7]. Recently, several animal experiments demonstrated that inhibition of either Rho or ROCK (Rho/ROCK) attenuated cardiomyopathy in diabetes and improved myocardial compliance [8–10]. Therefore, a Rho/ROCK inhibitor would be a good candidate for treating diabetes and its complications [7, 11, 12]. The first-generation Rho/ROCK inhibitor fasudil has been studied widely and applied in clinical practice [13]. The safety and efficacy of fasudil in treating pulmonary arterial hypertension and other cardiovascular and cerebrovascular diseases have been identified clearly in clinical trials [14–16]. However, few studies have focused on the effect of Rho/ROCK inhibitors on cardiac mitochondria *in vivo* or *in vitro*.

## 2. Materials and Methods

### 2.1. Animals

All experiments were performed according to the Guidelines of Animal Experiments from the Committee of Medical Ethics at the National Health Department of China and were approved by the Laboratory Center of Shanghai Tenth People's Hospital. Male Sprague Dawley rats that weighed 200 g were purchased from the Shanghai Slac Laboratory Animal Co., Ltd., and housed in plastic cages with well-ventilated stainless steel grid tops at room temperature with a 12-hour light/dark cycle.

### 2.2. Induction of Diabetes and Drug Treatment

 Type 2 diabetic (T2DM) rats were induced by a high-fat diet consisting of 42% fat (mainly pork), 19% protein, and 39% carbohydrate as well as a low dose of streptozotocin (STZ) (50 mg/kg intraperitoneally in a 0.1 mol/L citrate buffer, pH 4.5) as described previously [17]. Moreover, an oral glucose tolerance test (OGTT) was performed as described previously to confirm the onset of insulin resistance (IR) after eight weeks of dietary manipulation [17]. Diabetes was induced by a single intraperitoneal injection of STZ (Sigma, St. Louis, MO, USA) given to rats with insulin resistance.

One week after the initial STZ administration, rats with fasting blood glucose (FBG) ≥11.1 mmol/L in two consecutive analyses were considered to qualify as having T2DM. Subsequently, twenty diabetic rats were randomly divided into an untreated diabetic group (*n* = 10) and a fasudil-treated group (*n* = 10). Ten SD rats fed with a normal rat chow were considered as control group. The rats in the fasudil group were treated with fasudil (5 mg/kg bid) by intraperitoneal injection as reported previously [8], whereas untreated diabetic rats and control rats were injected intraperitoneally with equivalent volumes of saline for 4 weeks. All animals remained on the assigned diet until termination of the experiment. Fasudil hydrochloride was obtained from Chase Sun Pharmaceutical Co., Ltd. (Tianjin, China).

After four weeks of high-fat diet initiated at the time of fasudil administration, the FBG level was determined again. The rats were then anaesthetized by using 3% pentobarbital (30 mg/kg intraperitoneally), and plasma (8–10 mL per animal) was immediately collected from the femoral artery and processed into serum. After being washed in ice-cold saline solution, the hearts of the animals were weighed and frozen in liquid nitrogen then stored at −80°C.

### 2.3. Preparation of Cardiac Mitochondria

Mitochondria were isolated from rat hearts by differential centrifugation using a Tissue Mitochondria Isolation Kit (Thermo Scientific, MA, USA). After removal of the extraventricular tissue, the ventricle was weighed, finely minced in ice-cold buffer (160 mM KCl, 10 mM EGTA, and 0.5% fatty acid-free bovine serum albumin (BSA), pH 7.4), and brought to a final concentration of 1 g/10 mL of buffer. This tissue suspension was homogenized and centrifuged at 1,000 g for 10 min at 2°C. The supernatant was then centrifuged at 8,000 g for 10 min at 2°C to obtain the initial mitochondrial pellet, which was resuspended in suspension buffer (320 mM sucrose, 10 mM Tris-HCl, pH 7.4), and centrifuged again at 8,000 g for 10 min at 2°C to obtain the final mitochondrial pellet.

### 2.4. Ultrastructural Examination by Transmission Electron Microscopy (TEM)

Cardiac tissue was cut into approximately 1 mm^3^ pieces, fixed in 2.5% glutaraldehyde in 0.1 mol/L sodium phosphate buffer (pH 7.4) for 3 h at 4°C, and osmicated in 1% osmium tetroxide for 1 h at 4°C. After dehydration with a graded ethanol series, the sample was embedded in Epon812 and sectioned using a Leica EM UC6 (Leica Co., Vienna, Austria) ultramicrotome. Sections were viewed and photographed following TEM using a Tecnai G2 20 (FEI Co., Oregon, USA) at 200 kV.

### 2.5. Measurements of Succinic Dehydrogenase (SDH) and Monoamine Oxidase (MAO) in Cardiac Mitochondria

Each cardiac tissue sample was weighed to prepare a 10% (w/v) buffered homogenate (100 mg tissue/mL of 50 mM phosphate buffer at pH 7.2). The homogenate was centrifuged, and biochemical analyses were carried out using the supernatant. The protein concentration of the supernatant was determined by the Lowry method, using BSA as a standard. Activities of SDH and MAO in cardiac mitochondria were measured by spectrophotometry-based assays using commercially available kits (Jian Cheng Biological Engineering Institute, Nanjing, China).

### 2.6. Mitochondrial Permeability Transition Pore (MPTP) Opening in Cardiac Mitochondria

 Opening of MPTPs causes mitochondrial swelling [18]. Isolated cardiac mitochondria were resuspended in swelling buffer (120 mM KCl, 10 mM Tris-HCl, 20 mM MOPS 20, 5 mM KH_2_PO_4_ 5, pH 7.4) to a final concentration of 0.25 mg of mitochondrial protein/mL. After a 5 min equilibration period, swelling was induced by the addition of CaCl_2_ (200 *μ*M). The decrease at A520 was measured spectrophotometrically for 15 min.

### 2.7. JC-1 Staining and Mitochondrial Membrane Potentials Assay

 Loss of mitochondrial membrane potential was assessed using a fluorescence microscope (Leica DMI6000, Leica, Germany) following staining with 5,5′,6,6′-tetrachloro-1,1′,3,3′-tetraethylbenzimidazole-carbocyanide iodine (JC-1; Beyotime, China). Isolated cardiac mitochondria were stained with JC-1 staining solution (5 *μ*g/mL) for 15 min at 37°C and rinsed twice with phosphate buffer solution (PBS).

Mitochondrial membrane potentials were monitored by determining the relative amounts of dual emissions from mitochondrial JC-1 monomers or polymers using the fluorescent microscope. Mitochondrial depolarization was indicated by an increase in the red/green fluorescence intensity ratio. Red emission of the dye represented JC-1 polymers formed after potential-dependent aggregation in the mitochondria, reflecting normal mitochondrial membrane potential. Green fluorescence, which represented the monomeric form of JC-1, appeared in the cytosol after depolarization of the mitochondrial membrane.

The fluorescence was detected using a microplate reader (Tecan infinite 200). The wavelengths of excitation and emission used to detect the monomeric form of JC-1 were 514 nm and 529 nm, whereas 585 nm and 590 nm were used to detect polymers of JC-1. The ratio of JC-1 monomers and JC-1 polymers represented the condition of membrane potential of the cardiac mitochondria.

### 2.8. Protein Extraction and Western Blot Analysis

 Cardiac tissues (0.5 cm^3^) were homogenized in ice-cold radioimmunoprecipitation assay (RIPA) buffer and centrifuged at 10,000 g for 10 min at 4°C prior to collecting the supernatants. Protein concentrations in the supernatants were measured using the bicinchoninic acid (BCA) protein assay kit (Pierce Chemical Company, Rockford, USA). Proteins (40 *μ*g) were separated with 8% or 10% SDS-polyacrylamide gel electrophoresis and transferred to PVDF membranes, which were then washed with Tris-buffered saline, blocked with 5% skimmed milk powder (except for phosphorylated myosin phosphatase targeting protein (MYPT), which was blocked using 5% BSA) in Tris-buffered saline Tween-20 for 1 h, and incubated with the appropriate primary antibody at dilutions recommended by the supplier. The membrane was then washed, and primary antibodies were detected with secondary antibody conjugated to horseradish peroxidase for 1 h at room temperature. The blots were then developed with SuperSignal-enhanced chemiluminescent substrate solution (Pierce Chemical Company, Rockford, USA). Anti-*β*-actin, anti-ROCK 1, anti-ROCK 2, anti-RhoA, anti-p-MYPT and anti-MYPT antibodies were purchased from Cell Signaling Technology (Danvers, Massachusetts, USA).

### 2.9. Measurements of Biomarkers of Oxidative Stress

The supernatant collected after centrifugation of the cardiac tissue homogenate was used to determine the total antioxidant capacity (TAC), as well as malonyldialdehyde (MDA), hydroxy radical, reduced glutathione (GSH), and superoxide dismutase (SOD) levels using commercial kits (Jian Cheng Biological Engineering Institute, Nanjing, China).

### 2.10. Statistical Analysis

All values were expressed as mean ± SD. Data distributions were examined for normality and homogeneity of variance. Statistical significance was determined by one-way ANOVA with Newman-Keuls post hoc test. For nonparametric analysis, the Mann-Whitney *U* test with post hoc analysis was used to evaluate the difference among three groups. Values of *P* < 0.05 were considered significant.

## 3. Results

### 3.1. Effects of Fasudil on Diabetic Rats

Table 1 details the characteristics of three groups of rats at the end of the experimental period. The final body weights of rats were significantly higher in members of the fasudil-treated and control group compared with members of the diabetes group (all *P* < 0.05). The mean blood pressure (MBP) was a little lower in fasudil-treated rats than in diabetic and control rats, although the difference was not significant. Levels of blood glucose, serum insulin, glycated hemoglobin (HbA1c), and cholesterol were markedly elevated in fasudil-treated and untreated diabetic group compared with the control group (all *P* < 0.05). However, these values have no differences between diabetic rats treated with fasudil and untreated diabetic rats. Although the level of triglyceride was lower in control rats, there were no significant differences among three groups. The untreated diabetic group had significantly higher levels of free fatty acids compared to the other two groups (*P* < 0.05).

### 3.2. Changes in Mitochondrial Morphology

**Figure 1:**
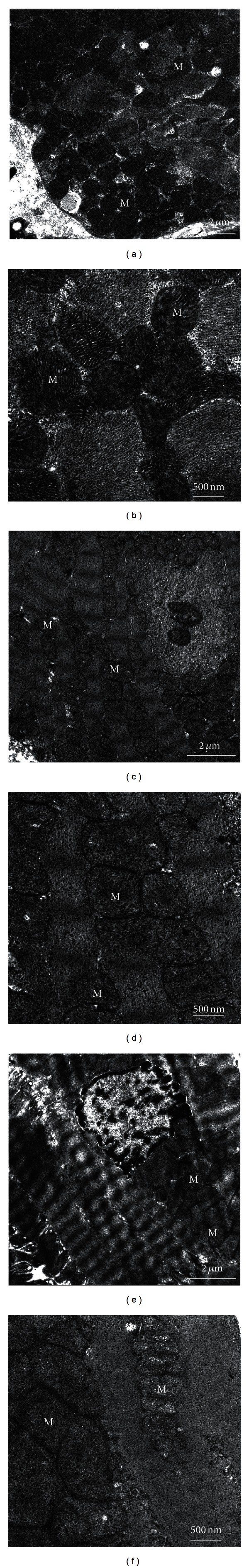
Effects of fasudil on the mitochondria of hearts from diabetic rats. The mitochondria of heart tissues were detected by TEM from the left ventricle of the rats. (a) Untreated diabetic group, 2,500x; (b) untreated diabetic group, 6,500x; (c) fasudil-treated diabetic group, 2,500x; (d) fasudil-treated diabetic group, 6,500x; (e) control group, 2,500x; (f) control group, 6,500x. Several mitochondria (M) exhibited swelling, enlargement, and diffuse edema, and disordered and partially disrupted mitochondrial cristae were observed in mitochondria from untreated diabetic rats. The damage to myocardial ultrastructures in fasudil-treated rats was attenuated compared with that of the untreated diabetic group.

We examined the mitochondrial morphology by TEM to confirm the cardioprotective effect of fasudil on mitochondrial biogenesis. Analysis using TEM indicated diffuse edema and enlargement in cardiac mitochondria of untreated diabetic rats, in which the mitochondrial cristae were disordered and partially disrupted (Figures 1(a) and 1(b)) compared with control group (Figures 1(e) and 1(f)). However, less damage to myocardial ultrastructures was observed in fasudil-treated rats compared with diabetic untreated rats (Figures 1(c) and 1(d)).

### 3.3. Changes in SDH and MAO Activity in Cardiac Mitochondria

**Figure 2:**
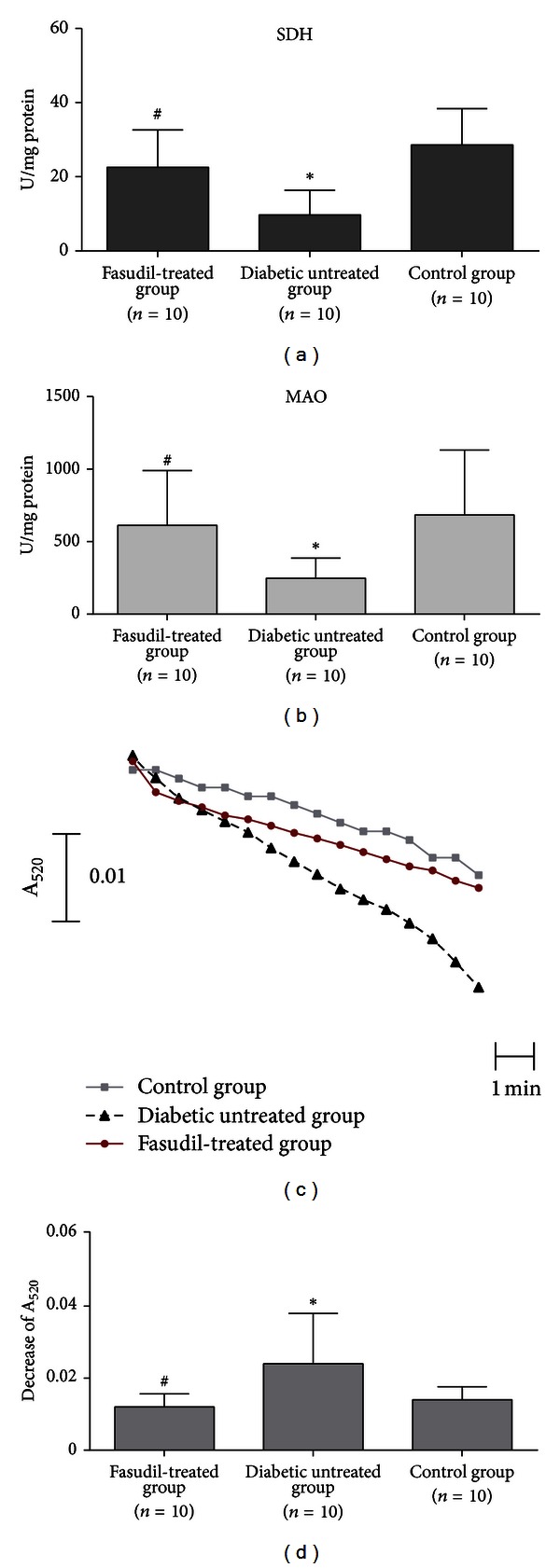
Comparison of SDH and MAO activities, MPTP opening, and decreases in A_520_ for the two groups. (a) Comparison of SDH activities in cardiac mitochondria from fasudil-treated, untreated diabetic, and control rats revealed statistically significant differences. SDH activities in cardiac mitochondria from fasudil-treated group were marked higher than that of untreated diabetic group (*P* = 0.003). (b) Comparison of MAO activities in cardiac mitochondria from fasudil-treated, untreated diabetic, and control rats revealed statistically significant differences. MAO activities in cardiac mitochondria from fasudil-treated group were significantly higher than that of untreated diabetic group (*P* = 0.015). (c) Effects of fasudil on MPTP opening (A_520_: absorbance at 520 nm). The trace represented the mean of A_520_ from three groups. The top trace represented cardiac mitochondria from SD control rats, the middle trace showed the absorbance in mitochondria from diabetic rats treated with fasudil, and lower traces showed the absorbance in mitochondria from untreated diabetic rats. (d) Comparison of decreases in A_520_ for the three groups. The decreases in A_520_ were significantly lower in diabetic rats treated with fasudil compared to untreated rats (*P* < 0.001).

Activities of SDH in fasudil-treated, untreated diabetic, and control rats were 22.8 ± 10.1, 9.8 ± 6.7, and 28.9 ± 9.8 U/mg protein, respectively. Activities of MAO in fasudil-treated group, diabetic-untreated group and control group were 617.9 ± 381.3, 250.4 ± 140.8, and 690.1 ± 451.6 U/mg protein, respectively. Activities of SDH and MAO were significantly higher in the cardiac mitochondria of fasudil-treated diabetic and control rats compared with those from untreated diabetic rats (all *P* < 0.05) (Figures 2(a) and 2(b)). These findings suggest that SDH and MAO activity in cardiac mitochondria could be restored by treatment with fasudil.

### 3.4. Effect of Fasudil on MPTP

 To investigate the degree of mitochondrial swelling, we assessed the opening of MPTP by measuring the absorbance of mitochondrial suspensions at 520 nm (A_520_) as reported previously [19, 20]. A decrease at A_520_ was considered to indicate swelling of the mitochondrion as a result of opening the pore. The decrease at A_520_ in the mitochondrial suspension was significantly attenuated in fasudil-treated rats compared with that of the mitochondrial suspension prepared from untreated rats (Figure 2(c)). The decrease of A_520_ in the mitochondrial suspension in fasudil-treated group, untreated diabetic group, and control group are 0.012 ± 0.0037, 0.024 ± 0.014, and 0.014 ± 0.0036, respectively. The decrease of A_520_ in fasudil-treated group is significantly lower than that of the untreated diabetic rats (*P* = 0.018) (Figure 2(d)). 

### 3.5. Changes in Mitochondrial Transmembrane Potential

**Figure 3:**
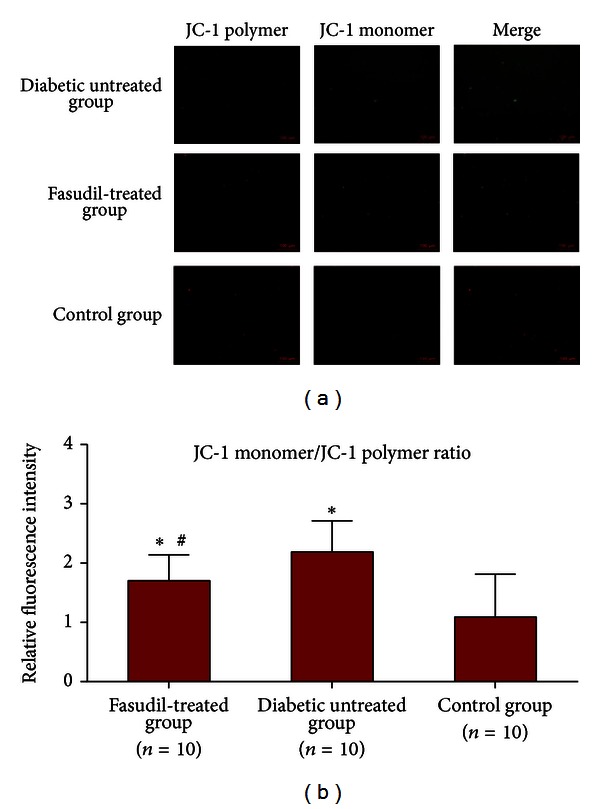
Effect of fasudil on transmembrane potential in mitochondria. Red fluorescence represents the mitochondrial aggregate form of JC-1 (JC-1 polymers), which indicates the intact mitochondrial membrane potential. Green fluorescence represents the monomeric form of JC-1 (JC-1 monomers), which indicates the dissipation of mitochondrial transmembrane potential. (a) Representative results of cardiac mitochondria stained with JC-1 and derived from fasudil-treated, untreated diabetic, and control rats. (b) Ratios of JC-1 monomer to JC-1 polymer (red/green fluorescence) for untreated diabetic rats, diabetic rats treated with fasudil, and control rats. Data are expressed as the mean ± SD. The ratio was significantly lower for diabetic rats treated with fasudil than for untreated diabetic rats (*P* = 0.037).

 JC-1 could aggregate in normal mitochondria and present red fluorescence. The dissipation of transmembrane potential in cardiac mitochondria isolated from diabetic-untreated rats was observed as increased green fluorescence (Figure 3(a)) following staining with JC-1. Consistent with its cardioprotective effects, fasudil moderated the dissipation of mitochondrial transmembrane potential. The ratio of red fluorescence to green fluorescence was also used to demonstrate the loss of mitochondrial transmembrane potential and the protective effect of fasudil in mitochondria.

In the fasudil-treated and control group, JC-1 aggregated in mitochondria and the JC-1 monomer/JC-1 polymer ratio were 1.72 ± 0.44 and 1.10 ± 0.73. A higher ratio in the untreated group (2.21 ± 0.53) indicated the dissipation of mitochondrial membrane potential. Diabetic rats treated with fasudil demonstrated attenuated dissipation of mitochondrial membrane potential (*P* < 0.05) (Figure 3(b)).

### 3.6. Rho/ROCK Activation in Cardiac Tissue and Rho/ROCK Suppression by Fasudil

**Figure 4:**
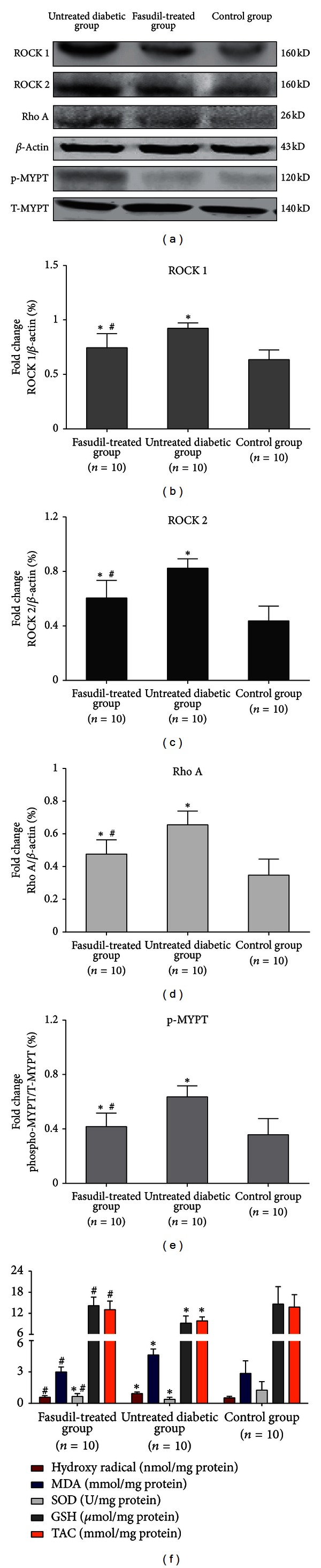
Effect of fasudil on cardiac Rho/ROCK activity. Levels of ROCK 1, ROCK 2, Rho A, and phosphorylated MYPT were detected in rat cardiac tissues using western blot analysis. (a) Representative results of assays of ROCK 1, ROCK 2, Rho A, and phospho-MYPT-1 and beta-actin abundances in rat cardiac tissues. (b) The levels of ROCK 1 protein expression were analyzed by western blot by using a polyclonal antibody to ROCK 1 to quantify its expressions in cardiac tissues. Data are expressed as the mean ± SD (*P* < 0.001 compared with untreated diabetic group). (c) The levels of ROCK 2 protein expression were analyzed by western blot by using a polyclonal antibody to ROCK 2 to quantify its expressions in cardiac tissues. Data are expressed as the mean ± SD (*P* < 0.001 compared with untreated diabetic group). (d) Average signal intensities quantified and expressed as percentage of the ratio of beta-actin to quantify Rho A expressions in cardiac tissues. Data are expressed as mean ± SD. The average signal intensities were significantly lower for diabetic rats treated with fasudil than for untreated diabetic rats (*P* = 0.008). (e) Average signal intensities quantified and expressed as a percentage of the ratio of T-MYPT to quantify levels of phosphorylated MYPT in cardiac tissues. Data are expressed as the mean ± SD. The ratio was significantly lower for diabetic rats treated with fasudil than for untreated diabetic rats (*P* = 0.019). (f) Five oxidative stress biomarkers were measured in heart tissues from diabetic-treated, untreated, and control rats. *P < 0.05 versus control group. ^#^ *P* < 0.05 versus untreated diabetic group.

 To investigate the protein expression of ROCK 1, ROCK 2, Rho A, and MYPT, we isolated rat cardiac tissue and performed western blot analysis. These proteins were expressed in the heart tissue of diabetic rats. Moreover, to examine the efficacy of fasudil for Rho/ROCK inhibition in the cardiac tissue, we quantified the amount of phosphorylated MYPT (p-MYPT) and total MYPT. Both phosphorylated and unphosphorylated MYPT are downstream targets of ROCK. As shown in Figure 4(a), levels of ROCK 1, ROCK 2, Rho A, and phosphorylated MYPT were markedly higher in the cardiac tissue of diabetic untreated rats than in the cardiac tissue from fasudil-treated rats (0.95 ± 0.06; 0.57 ± 0.11; 0.69 ± 0.10, *P* < 0.05 versus fasudil-treated group). In comparison, treatment of diabetic rats with fasudil significantly reduced the levels of ROCK 1, ROCK 2, Rho A, and p-MYPT (0.75 ± 0.11; 0.44 ± 0.093; 0.55 ± 0.13) (Figures 4(b), 4(c), and 4(d)).

### 3.7. Changes in Oxidative Stress Biomarkers

The levels of oxidative stress biomarkers in cardiac tissues of the two groups were significantly different (all *P* < 0.05) (Table 2).  Figure 4(e) shows the comparison of TAC, MDA, hydroxy radical, GSH, and SOD levels in cardiac tissues from the two groups of diabetic rats. The levels of TAC, GSH, and SOD were significantly lower in the untreated diabetic rats than in fasudil-treated rats, whereas the levels of MDA and hydroxy radical were significantly higher in diabetic untreated rats compared with diabetic rats treated with fasudil (*P* < 0.001).

## 4. Discussion

The main aim of this study was to determine whether the Rho/ROCK inhibitor fasudil could protect cardiac mitochondria from diabetic rats fed a combination of a low-dose STZ and a high-fat diet. The effect of fasudil on both the structure and function of cardiac mitochondria were investigated. These data complement findings from our previous study, which suggested that inhibition of Rho/ROCK signaling may have therapeutic potential in preventing diabetes associated with vascular inflammation and atherogenesis [21].

The main function of cardiac mitochondria is to generate adenosine triphosphate (ATP) through oxidative phosphorylation (Ox-Phos). Under normal conditions, the adult heart relies mostly on fatty acids to fuel Ox-Phos, with 10% to 30% of total ATP derived from glucose [22]. It is believed that altered mitochondrial bioenergetics appear to play a substantial role in cardiomyopathy or myocardial dysfunction [23]. T2DM is one of the most common metabolic diseases in the world, and it is associated with an elevated rate of oxidative stress [24]. Reactive oxygen species (ROS) produced by mitochondria have been implicated in the pathogenesis of T2DM and its complications [25–27]. Therefore, mitochondrial defects may play a critical role in pathogens and development of T2DM [28], and the protection of mitochondria could be considered as a potential treatment target for diabetes.

Xie and colleagues [6] reported that diabetes-associated ROS or lipoproteins impair mitochondrial respiration. Reduced activities of SDH and other key mitochondrial enzymes in diabetic animals potentially lead to oxidative stress and the development of diabetic cardiovascular complications. The key respiratory enzyme SDH, which is assembled in mitochondria to form the mitochondrial complex 2, links the Krebs cycle to the electron transport chain. Recent reports have highlighted the relevance of MAO to the formation of mitochondrial ROS formation [29, 30]. 

The MPTP is a common target of intracellular signal transduction pathways. Opening this pore allows water and solutes to enter the mitochondria, increases the matrix volume, and ruptures the outer mitochondrial membrane, leading to the release of intermembranous cytochrome C. Thus, MPTP was considered to play an important role in cell death and apoptosis [31, 32]. The accumulation of mitochondrial ROS induced by hyperglycemia or diabetes could dissipate mitochondrial transmembrane potential and activate MPTP directly. Dissipation of mitochondrial transmembrane potential also represented mitochondrial dysfunction. Our study showed that the opening of the MPTP was inhibited by fasudil in diabetic rat hearts. In addition, diabetic rats treated with fasudil attenuated the dissipation of mitochondrial transmembrane potential (*P* = 0.037). This indicated that the Rho/ROCK inhibitor fasudil could exert a cardioprotective effect through the inhibition of MPTP and the dissipation of mitochondrial transmembrane potential.

The Rho/ROCK pathway may associate with enhanced oxidative stress by upregulation of NAD(P)H oxidase [33]. Furthermore, it has been reported that fasudil exerted antioxidant effects on hypercholesterolemic rats [34]. We speculate that fasudil has similar effects on cardiac tissue and plays a protective role in cardiac mitochondria. Therefore, several oxidative stress biomarkers (TAC, MDA, hydroxy radical, GSH, and SOD) in cardiac tissue, as well as SDH and MAO activities in cardiac mitochondria were measured in this study. Our study demonstrated that TAC, GSH, and SOD in cardiac tissue and SDH and MAO activities in cardiac mitochondria were significantly higher in fasudil-treated rats than in untreated rats (*P* < 0.001). Additionally, the levels of MDA and hydroxy radicals in cardiac tissue were significantly higher in untreated diabetic rats (*P* < 0.001).

Our findings might have implications regarding the efficacy of the administration of fasudil to treat diabetic cardiomyopathy or diabetic cardiovascular complications. A previous study demonstrated that fasudil might have potential benefits on cardiac function in diabetic rats [8]. Our results further suggest that fasudil is likely to have a cardioprotective effect by protecting cardiac mitochondria, because the structure and function of cardiac mitochondria in fasudil-treated rats were significantly higher than those observed in untreated rats.

## 5. Conclusions

In summary, we found that not only can the cardiac mitochondrial structure be improved by administration of Rho/ROCK inhibitor fasudil to T2DM rats, but the SDH and MAO activities in cardiac mitochondria were significantly higher in fasudil-treated rats than in untreated rats. These beneficial effects may be associated with the ability of fasudil treatment to attenuate oxidative stress.

## Figures and Tables

**Table 1:** Effects of fasudil on body weight, heart weight, blood pressure, blood glucose, serum insulin, glycated hemoglobin, and lipid profiles in diabetic rats either treated with or without fasudil.

	Fasudil-treated diabetic group (*n* = 10)	Untreated diabetic group (*n* = 10)	Control group (*n* = 10)
Start weight (g)	191 ± 7	190 ± 8	192 ± 7
End weight (g)	465 ± 9^#^	442 ± 10*	465 ± 10
Heart weight (g)	1.72 ± 0.11	1.68 ± 0.09*	1.78 ± 0.06
Mean blood pressure (kpa)	8.21 ± 1.21	9.44 ± 1.59	8.43 ± 1.25
Fasting blood glucose (mmol/L)	20.0 ± 2.6*	19.9 ± 2.4*	6.1 ± 0.2
Serum insulin (ng/mL)	6.17 ± 0.99*	5.97 ± 1.00*	4.78 ± 0.67
Glycated hemoglobin (%)	13.4 ± 1.7*	12.5 ± 1.8*	4.9 ± 0.4
Total cholesterol (mmol/L)	1.41 ± 0.15*	1.40 ± 0.13*	0.95 ± 0.12
Triglyceride (mmol/L)	0.63 ± 0.10	0.61 ± 0.08	0.55 ± 0.06
Free fatty acids (mmol/L)	0.33 ± 0.06	0.40 ± 0.18*	0.28 ± 0.07

Data are presented as the mean ± SD.

*P < 0.05 versus control group.

^#^ *P* < 0.05 versus untreated diabetic group.

**Table 2:** Comparison of oxidative stress biomarkers in diabetic rats either treated with or without fasudil.

	Fasudil-treated diabetic group (*n* = 10)	Untreated diabetic group (*n* = 10)	Control group (*n* = 10)
TAC (mmol/mg protein)	13.03 ± 2.46^#^	9.77 ± 1.14*	13.76 ± 3.60
MDA (mmol/mg protein)	3.02 ± 0.49^#^	4.65 ± 0.57*	2.88 ± 1.21
hydroxy radical (nmol/mg protein)	0.59 ± 0.15^#^	0.94 ± 0.15*	0.53 ± 0.14
GSH (*μ*mol/mg protein)	14.16 ± 2.47^#^	9.13 ± 2.06*	14.61 ± 5.07
SOD (U/mg protein)	0.66 ± 0.28^#∗^	0.38 ± 0.20*	1.27 ± 0.83

Data are presented as the mean ± SD.

*P < 0.05 versus control group.

^#^ *P* < 0.05 versus untreated diabetic group.

## References

[1] McBride HM, Neuspiel M, Wasiak S (2006). Mitochondria: more than just a powerhouse. *Current Biology*.

[2] Lesnefsky EJ, Moghaddas S, Tandler B, Kerner J, Hoppel CL (2001). Mitochondrial dysfunction in cardiac disease: ischemia—reperfusion, aging, and heart failure. *Journal of Molecular and Cellular Cardiology*.

[3] Walters AM, Porter GA, Brookes PS (2012). Mitochondria as a drug target in ischemic heart disease and cardiomyopathy. *Circulation Research*.

[4] Shen GX (2012). Mitochondrial dysfunction, oxidative stress and diabetic cardiovascular disorders. *Cardiovascular & Hematological Disorders—Drug Targets*.

[5] Shen GX (2010). Oxidative stress and diabetic cardiovascular disorders: roles of mitochondria and NADPH oxidase. *Canadian Journal of Physiology and Pharmacology*.

[6] Xie X, Chowdhury SR, Sangle G, Shen GX (2010). Impact of diabetes-associated lipoproteins on oxygen consumption and mitochondrial enzymes in porcine aortic endothelial cells. *Acta Biochimica Polonica*.

[7] Arita R, Hata Y, Nakao S (2009). Rho kinase inhibition by fasudil ameliorates diabetes-induced microvascular damage. *Diabetes*.

[8] Guan SJ, Ma ZH, Wu YL (2012). Long-term administration of fasudil improves cardiomyopathy in streptozotocin-induced diabetic rats. *Food and Chemical Toxicology*.

[9] Zhou H, Li YJ, Wang M (2011). Involvement of RhoA/ROCK in myocardial fibrosis in a rat model of type 2 diabetes. *Acta Pharmacologica Sinica*.

[10] Kizub I, Pavlova O, Johnson C, Soloviev A, Zholos A (2010). Rho kinase and protein kinase C involvement in vascular smooth muscle myofilament calcium sensitization in arteries from diabetic rats. *British Journal of Pharmacology*.

[11] Bach LA (2008). Rho kinase inhibition: a new approach for treating diabetic nephropathy?. *Diabetes*.

[12] Komers R (2011). Rho kinase inhibition in diabetic nephropathy. *Current Opinion in Nephrology and Hypertension*.

[13] Ishida T, Takanashi Y, Kiwada H (2006). Safe and efficient drug delivery system with liposomes for intrathecal application of an antivasospastic drug, fasudil. *Biological and Pharmaceutical Bulletin*.

[14] Omeis I, Jayson NA, Murali R, Abrahams JM (2008). Treatment of cerebral vasospasm with biocompatible controlled-release systems for intracranial drug delivery. *Neurosurgery*.

[15] Olson MF (2008). Applications for ROCK kinase inhibition. *Current Opinion in Cell Biology*.

[16] Suzuki Y, Shibuya M, Satoh SI, Sugimoto Y, Takakura K (2007). A postmarketing surveillance study of fasudil treatment after aneurysmal subarachnoid hemorrhage. *Surgical Neurology*.

[17] Reed MJ, Meszaros K, Entes LJ (2000). A new rat model of type 2 diabetes: the fat-fed, streptozotocin-treated rat. *Metabolism: Clinical and Experimental*.

[18] Baines CP, Song CX, Zheng YT (2003). Protein kinase C*ε* interacts with and inhibits the permeability transition pore in cardiac mitochondria. *Circulation Research*.

[19] Javadov S, Karmazyn M, Escobales N (2009). Mitochondrial permeability transition pore opening as a promising therapeutic target in cardiac diseases. *Journal of Pharmacology and Experimental Therapeutics*.

[20] Gao Q, Zhang SZ, Cao CM, Bruce IC, Xia Q (2005). The mitochondrial permeability transition pore and the Ca^ 2+^-activated K^ +^ channel contribute to the cardioprotection conferred by tumor necrosis factor-*α*. *Cytokine*.

[21] Li H, Peng W, Jian W (2012). ROCK inhibitor fasudil attenuated high glucose-induced MCP-1 and VCAM-1 expression and monocyte-endothelial cell adhesion. *Cardiovascular Diabetology*.

[22] Opie LH (1971). Substrate utilization and glycolysis in the heart. *Cardiology*.

[23] Huss JM, Kelly DP (2005). Mitochondrial energy metabolism in heart failure: a question of balance. *The Journal of Clinical Investigation*.

[24] Piarulli F, Sartore G, Lapolla A (2012). Glyco-oxidation and cardiovascular complications in type 2 diabetes: a clinical update. *Acta Diabetologica*.

[25] Zhang DX, Gutterman DD (2007). Mitochondrial reactive oxygen species-mediated signaling in endothelial cells. *American Journal of Physiology*.

[26] Giacco F, Brownlee M (2010). Oxidative stress and diabetic complications. *Circulation Research*.

[27] Folli F, Corradi D, Fanti P (2011). The role of oxidative stress in the pathogenesis of type 2 diabetes mellitus micro- and macrovascular complications: avenues for a mechanistic-based therapeutic approach. *Current Diabetes Reviews*.

[28] Lowell BB, Shulman GI (2005). Mitochondrial dysfunction and type 2 diabetes. *Science*.

[29] Di Lisa F, Kaludercic N, Carpi A, Menabò R, Giorgio M (2009). Mitochondrial pathways for ROS formation and myocardial injury: the relevance of p66Shc and monoamine oxidase. *Basic Research in Cardiology*.

[30] Di Lisa F, Kaludercic N, Carpi A, Menabò R, Giorgio M (2009). Mitochondria and vascular pathology. *Pharmacological Reports*.

[31] Halestrap AP, Clarke SJ, Javadov SA (2004). Mitochondrial permeability transition pore opening during myocardial reperfusion—a target for cardioprotection. *Cardiovascular Research*.

[32] Weiss JN, Korge P, Honda HM, Ping P (2003). Role of the mitochondrial permeability transition in myocardial disease. *Circulation Research*.

[33] Higashi M, Shimokawa H, Hattori T (2003). Long-term inhibition of Rho-kinase suppresses angiotensin II-induced cardiovascular hypertrophy in rats in vivo: effect on endothelial NAD(P)H oxidase system. *Circulation Research*.

[34] Ma Z, Zhang J, Ji E, Cao G, Li G, Chu L (2011). Rho kinase inhibition by fasudil exerts antioxidant effects in hypercholesterolemic rats. *Clinical and Experimental Pharmacology and Physiology*.

